# Folate Status Shaped by Taste Receptor Genetics and Sociobehavioral Modulation: Evidence from a Hungarian Cohort

**DOI:** 10.3390/nu18040562

**Published:** 2026-02-08

**Authors:** Peter Piko, Judit Dioszegi, Nora Kovacs, Roza Adany

**Affiliations:** 1 Department of Public Health and Epidemiology, Faculty of Medicine, University of Debrecen, 4032 Debrecen, Hungary; dioszegi.judit@med.unideb.hu (J.D.); or adany.roza@semmelweis.hu (R.A.); 2 National Laboratory for Health Security, Center for Epidemiology and Surveillance, Semmelweis University, 1089 Budapest, Hungary; 3 HUN-REN-UD Public Health Research Group, Department of Public Health and Epidemiology, Faculty of Medicine, University of Debrecen, 4032 Debrecen, Hungary; 4 Institute of Preventive Medicine and Public Health, Semmelweis University, 1089 Budapest, Hungary

**Keywords:** polygenic score, taste receptor genes, nutrigenetics, dietary behaviors, serum folate concentration, sociodemographic factors, vegetable intake, Roma population, structural equation modeling

## Abstract

Background: Folate is essential for one-carbon metabolism, yet deficiency remains common in non-fortified populations. Bitter-taste-receptor genetics may influence vegetable intake and thus folate status, but the cumulative impact of sensory genetics, diet, and sociodemographic factors is unclear. This study aimed to investigate how taste-related genetic variants, aggregated into a polygenic score (PGS), together with dietary behavior and sociodemographic factors, modulate serum folate levels in a Hungarian adult population, including Roma ethnic minority participants. Methods: In a cross-sectional sample of 626 adults (312 from the Hungarian general population and 314 from the Roma ethnic minority), serum folate was quantified by chemiluminescent immunoassay, and eight taste-related single-nucleotide polymorphisms (SNPs) were genotyped. A four-SNP PGS (*TAS2R19* rs10772420, *OR10G4* rs1527483, *TRPV1* rs8065080, and *CD36* rs1761667) was optimized via the stepwise method (ΔR^2^ criterion, FDR q < 0.05). Multivariable linear regression was used to assess associations with continuous folate, and logistic models were used to evaluate deficiency risk (≤13 µmol/L; area under the curve, AUC). Interaction terms were tested for effect modification by education and vegetable intake, and mediation pathways were examined by structural equation modeling with 1000 bootstrap replications. Results: *TAS2R19* rs10772420 was found to be the strongest predictor of serum folate level. This effect remained significant even after adjusting for vegetable intake (β = 1.12 nmol/L; *p* = 0.003), suggesting a persistent genetic association independent of vegetable intake. The taste-related PGS exhibited a significant dose–response relationship with folate levels (*p* < 0.001) but had only modest discriminatory power for deficiency (AUC = 0.569). Higher educational attainment amplified the associations between the PGS and folate levels (*p* for interaction < 0.05), whereas vegetable intake did not mediate genetic effects. The associations were consistent across Hungarian general and Roma population subgroups. Conclusions: Bitter-taste-receptor genetics are associated with serum folate levels in a pattern not substantially mediated by self-reported vegetable intake, and this influence is further modified by education. These findings support the development of genome-informed, culturally tailored nutrition strategies for non-fortified populations.

## 1. Introduction

Folate (vitamin B9) is a water-soluble micronutrient essential for DNA synthesis, methylation, and cellular division [1,2]. Adequate folate status is particularly critical during pregnancy, as deficiency is strongly associated with neural tube defects (NTDs) and other developmental disorders [3,4,5]. In addition to its role in fetal development, folate contributes to cardiovascular, neurological, and mental health by being involved in homocysteine metabolism and neurotransmitter synthesis [6,7,8].

Globally, folate deficiency remains a significant public health concern, particularly in low- and middle-income countries [9,10]. While mandatory folic acid fortification has reduced deficiency rates in countries like the US and Canada [11,12], most European nations [13], including Hungary, rely on voluntary supplementation [14]. Consequently, folate intake and status across Europe exhibit significant heterogeneity, with suboptimal levels reported in specific populations, notably among women of reproductive age [15,16]. In Hungary, where folic acid fortification is not mandatory [17], there is a unique opportunity to explore folate status from multiple aspects, such as dietary behavior and genetic conditions.

The status of folate in the human body is influenced by a complex interplay of environmental, lifestyle, and genetic factors [18]. Dietary intake is clearly the primary determinant, with leafy greens, legumes, and fortified grains serving as key sources [19]. However, it is important to note that the absorption and metabolism of folate are modulated by genetic polymorphisms in enzymes such as methylenetetrahydrofolate reductase (*MTHFR*) [20], folate hydrolase 1 (*FOLH1*), and dihydrofolate reductase (*DHFR*) [21]. These genes can affect bioavailability, transport, and intracellular retention of folate, thereby contributing to interindividual variability in serum and red blood cell folate concentrations.

As demonstrated in a previous study [22], synthetic folic acid is a stronger determinant of circulating folate levels than naturally occurring food folate, particularly in populations with mandatory fortification. Conversely, in countries such as Hungary, where fortification is limited, dietary preferences—potentially influenced by genetic taste sensitivity [23]—may exert a more substantial influence on folate status. Furthermore, gene–diet interactions, such as those involving *FOLH1* [24], underscore the necessity of considering both genetic and behavioral factors when analyzing folate metabolism. The present findings extend this paradigm by demonstrating that a genetic risk score related to taste is associated with serum folate levels [25]. This suggests that sensory perception may influence micronutrient status through dietary behavior.

Recent research has also explored the behavioral dimension of folate intake [26], particularly the role of taste perception in shaping dietary preferences. Single-nucleotide polymorphisms (SNPs) in taste receptor genes [25], including taste receptor type 2 member 38 (*TAS2R19*), taste receptor type 2 member 16 (*TAS2R16*), and transient receptor potential vanilloid 1 (*TRPV1*), have been associated with sensitivity to bitter and pungent compounds. For example, *TAS2R19* variants linked to heightened bitter sensitivity may lead individuals to avoid folate-rich vegetables such as broccoli and spinach. Lucock et al. [26] emphasized the role of bitter-taste sensitivity in the dietary avoidance of folate-rich vegetables, and Cummings et al. [22] examined metabolic gene scores in populations consuming fortified foods.

Despite the growing interest in nutrigenetics, the relationship between genetic variants related to taste and folate status remains underexplored. Polygenic scores (PGSs) for folate levels, which aggregate the effects of multiple single SNPs, offer a promising approach to quantifying the cumulative genetic influence on nutrient status.

The present study investigates the individual and combined effects of taste-related SNPs on serum folate concentrations in a general adult Hungarian population. The aggregated genetic influence is assessed using a PGS derived from the relevant SNPs.

A key objective is to examine whether vegetable consumption mediates the relationship between taste-related genetic variation and folate status. In addition, the analysis explores whether sociodemographic factors, such as education level and ethnicity, modify these associations.

By disentangling individual and polygenic effects and their behavioral correlates, this research contributes to the growing field of nutrigenetics and supports the development of genome-informed dietary strategies.

## 2. Materials and Methods

### 2.1. Study Populations

A detailed description of the study design and the data collection was published previously [27]. Briefly, participants representing the Hungarian general population were selected through the General Practitioners’ Morbidity Sentinel Stations Programme (GPMSSP), a registry established in 1998 to monitor major noncommunicable diseases [28]. Individuals aged 20–64 living in households in Borsod–Abaúj–Zemplén and Szabolcs–Szatmár–Bereg counties were randomly chosen from GP records. Although the initial plan was to recruit 25 individuals from each of 20 GP practices, two practices declined participation, resulting in a final sample of 450 individuals from 18 practices.

Participants from the Roma ethnic minority aged 20–64 years were selected using a stratified multistage sampling method in the same counties (Hajdú–Bihar and Szabolcs–Szatmár–Bereg) where Roma communities are densely located in segregated colonies. Colonies with more than 100 residents were identified during a prior environmental survey [29]. Ethnicity was self-reported. Twenty colonies were randomly selected from a verified database, followed by the random selection of 25 households per colony.

To enhance cultural sensitivity and data reliability, the household surveys in Roma communities were conducted by Roma interviewers undergoing university-level public health training. The inclusion criteria for both populations were an age range of 20–64 years, residence in the selected counties, and the ability to provide informed consent. Exclusion criteria included pregnancy, recent hospitalization, or an inability to complete the questionnaire. To address potential confounding from conditions affecting folate metabolism, we systematically assessed: (1) C-reactive protein (CRP), a marker of systemic inflammation; (2) alcohol consumption frequency; and (3) use of commonly prescribed medications (antihypertensive, antidiabetic, and lipid-lowering). In total, 832 individuals were enrolled: 417 from the general population and 415 from the Roma ethnic population.

Following rigorous quality control procedures, including the exclusion of participants with incomplete basic characteristics, genotyping failures, and protocol violations, a final analytic sample of 626 participants (75.2% of the enrolled cohort) was retained for statistical analysis. This final sample comprised 312 individuals from the Hungarian general population and 314 individuals from the Roma ethnic minority.

### 2.2. Power Calculation

An a priori power analysis was conducted using G*Power version 3.1 (Heinrich Heine University Düsseldorf, Düsseldorf, Germany) to determine the required sample size for a linear multiple regression model with 17 predictors. Based on an estimated effect size of *f*^2^ = 0.244, a significance level of 0.05, and a desired power of 0.80, the minimum required sample size was found to be 96. For a more stringent power of 0.95, the required sample size increased to 134. Given our actual sample size of 626, the study was well-powered to detect medium-to-large effects, ensuring reliable identification of significant predictors.

### 2.3. Ethical Approval

All participants provided written informed consent prior to enrollment. Ethical approval was granted by the tenets of the Declaration of Helsinki, and the protocol was approved by the Ethics Committee of the Hungarian Scientific Council for Health (No.: 61327–3/2017/EKU).

### 2.4. Data Collection

#### 2.4.1. Questionnaire-Based Data Collection

The study employed a standardized three-pillar methodology, comprising questionnaire-based interviews, physical examinations, and laboratory testing, which has previously been used to investigate cardiometabolic risk profiles, including the prevalence of metabolic syndrome [30,31] and insulin resistance in these populations [27].

This three-pillar survey design was developed to support a wide range of epidemiological and genetic association studies, enabling robust comparisons to be made between the Hungarian general population and the Roma minority. The protocol was based on internationally harmonized standards, including the European Health Interview Survey (EHIS) and the European Health Examination Survey (EHES), to ensure methodological consistency and cross-study comparability. The laboratory panel was selected to capture key cardiometabolic and inflammatory biomarkers, while the inclusion of DNA extraction enabled downstream genotyping and polygenic risk modeling.

Questionnaire-based interviews: Participants completed the EHIS Wave 2 questionnaire, supplemented with additional validated question sets on lifestyle, socioeconomic status, and health determinants. These included items such as the 12-Item General Health Questionnaire (GHQ-12), which has previously been used in national and international surveys.

#### 2.4.2. Physical Examination

Standardized physical measurements were performed according to the European Health Examination Survey (EHES) protocol. Height, weight, and waist circumference were measured using calibrated instruments, and body mass index (BMI) was calculated as kg/m^2^. Blood pressure was measured in a seated position after a minimum 5 min rest using validated automated sphygmomanometers. All measurements were taken by trained healthcare personnel following harmonized procedures.

#### 2.4.3. Laboratory Tests

After an overnight fast of at least 10 h, venous blood samples were collected into plain (serum separator) and EDTA-anticoagulated tubes. Samples were processed within 2 h, centrifuged, aliquoted, and stored at −80 °C until analysis. Serum folate concentrations were quantified using the ARCHITECT Folate assay (Abbott ARCHITECT i2000SR; reagent kit #01P7427, Abbott Laboratories, Abbott Park, IL, USA), a chemiluminescent microparticle immunoassay (CMIA) employing folate-binding protein-coated microparticles and acridinium-labelled folate analogues.

Serum folate categories were defined using a data-driven approach based on the distribution of folate concentrations in the study population. Three categories were created: <7 µmol/L (low), 7–13 µmol/L (normal), and >13 µmol/L (optimal). The <7 µmol/L threshold corresponds to the recognized biochemical indicator of folate deficiency [32], while the remaining cut-points reflect the central and upper segments of the cohort’s folate distribution. These categories were used for descriptive and trend analyses and do not represent clinical diagnostic thresholds.

The biochemical panel included the following fasting laboratory parameters measured from serum or plasma: fasting glucose, HbA1c, insulin, total cholesterol, HDL-cholesterol, LDL-cholesterol, triglycerides, apolipoprotein A1, apolipoprotein B100, creatinine, uric acid, C-reactive protein (CRP), and liver enzymes, including alanine aminotransferase (ALT), aspartate aminotransferase (AST), gamma-glutamyl transferase (GGT), and alkaline phosphatase (ALP).

Genomic DNA was extracted from EDTA-treated whole blood using the MagNA Pure LC DNA Isolation Kit—Large Volume (Roche Diagnostics, Mannheim, Germany; Cat. No. 03310515001). DNA concentration and purity were assessed by spectrophotometry (A260/A280 ratio).

### 2.5. Food and Taste Preference Assessment

Fruit and vegetable intake was assessed using the standardized EHIS Wave 2 frequency question [23] (“How often do you usually eat fruits/vegetables?”), which offered six ordered response categories ranging from “never” and “less than once a week” to “1–2 days per week”, “3–4 days per week”, “5–6 days per week”, and “every day”. To ensure consistency and to improve statistical power, these responses were subsequently collapsed into two groups: individuals consuming fruits or vegetables three or more times per week (corresponding to the original EHIS categories of 3–4 days per week, 5–6 days per week, and every day) and those consuming them less than three times per week (never, less than once a week, or 1–2 days per week).

Sugar intake was evaluated using the EHIS item asking participants how many teaspoons of sugar they typically added to foods or beverages per day, with response options ranging from “none” to “1–2 teaspoons”, “3–4 teaspoons”, and “five or more teaspoons”.

Salt use was assessed with the standard EHIS question on whether participants usually added salt to their food before tasting it, with a binary yes/no response format.

Taste preferences were assessed using a validated five-point Likert scale (response options: 1—strongly dislike, 2—dislike, 3—neutral, 4—like, 5—strongly like), adapted from earlier work in sensory perception research [33]. This scale covered sweet, fatty, salty, and bitter flavors, with specific food examples provided for each category. Bitter taste perception was explored through consumption of specific foods known to vary by *TAS2R* receptor genotype, including bitter chocolate, raw vegetables (e.g., broccoli, cabbage, and kohlrabi), and grapefruit. The specific food items and their selection rationale were previously documented in our earlier study [23], where the same taste preference assessment was employed with this Hungarian population. In that prior study, the taste preference items were developed based on a systematic review of genetic taste variant-phenotype associations [25], and reliability and construct validity of the taste preference items were demonstrated through assessment in the same cohort, ensuring consistency and validity in the current study.

For the purposes of the analysis, responses regarding taste preferences were grouped into two categories: ‘likes’ (combining responses 4–5: “like” and “strongly like”) and ‘dislikes or neutral’ (combining responses 1–3: “strongly dislike”, “dislike”, and “neutral”). This dichotomization was implemented to improve statistical power in regression models and to provide a clear and interpretable distinction between the two preference categories.

Fruit and vegetable consumption was categorized into two groups: individuals who consumed fruit or vegetables three or more times per week and those who consumed them less than three times per week.

### 2.6. Selection of Genetic Variants

The systematic literature review used to identify taste-preference-related SNPs was conducted and published previously [25]. Based on this study, nine SNPs associated with salty, sweet, fatty, and bitter tastes, as well as related food preferences, were identified. One SNP (SCNN1B rs3785368) was excluded due to genotyping quality control failures, specifically low call rate (<90%) and deviation from Hardy–Weinberg equilibrium. The final SNP list included the following polymorphisms: *TAS1R3* (rs307355), *CD36* (rs1761667 and rs1527483), *SCNN1B* (rs239345), *TRPV1* (rs8065080), *TAS2R38* (rs713598), *TAS2R19* (rs10772420), and *CA6* (rs2274333). For more details, see Table 1.

### 2.7. DNA Extraction and Genotyping

DNA was isolated from EDTA-anticoagulated blood samples using the MagNA Pure LC DNA Isolation Kit—Large Volume (Cat. No. 03310515001; Roche Diagnostics, Basel, Switzerland) by following the manufacturer’s protocol.

Genetic analysis was conducted at the Mutation Analysis Core Facility of Karolinska University Hospital (Stockholm, Sweden) using the MassARRAY platform with iPLEX Gold Chemistry. Quality control procedures included: (1) genotyping success rate assessment (call rate; threshold > 90%), (2) Hardy–Weinberg equilibrium (HWE) testing (*p* > 0.05) for each SNP, (3) allele frequency validation, and (4) signal-to-noise ratio evaluation for probe amplification quality. Overall genotyping success rate exceeded 98%, with validation and concordance checks performed according to standard protocols. One SNP (SCNN1B rs3785368) failed quality control criteria (call rate < 95% and HWE deviation) and was excluded from further analysis.

### 2.8. Polygenic Score Calculation

The construction of the polygenic score followed standard additive, allele-count-based PGS methodology as described in previous methodological work [43,44]. The individual effects of SNPs were examined using adjusted linear regression analysis that included all SNPs within a single model. This approach was employed to avoid the potential inflation of type I error that may result from analyzing the SNPs separately. Significant associations with folate concentration, treated as a continuous outcome variable, were identified.

The PGS was built from four SNPs selected according to:

- Strength of individual association (standardized regression coefficient);
- Incremental explained variance (ΔR^2^);
- Balanced allele weighting to maintain score stability.

Each SNP was coded under an additive genetic model (0 = homozygous non-risk, 1 = heterozygous, 2 = homozygous risk). The PGS is equal to the sum of these coded allele counts.

All SNPs included in the polygenic score were tested for Hardy–Weinberg equilibrium (HWE) using chi-square tests. No significant deviations were observed (*p* > 0.05), supporting the validity of genotype distributions in the study population.

Stepwise regression was then used to optimize the polygenic score by evaluating individual SNP effect sizes (standardized β coefficients), incremental improvements in model fit (ΔR^2^), and allele balance. The final model included four SNPs based on their consistent association with serum folate concentration. Linkage disequilibrium (LD) between the four SNPs included in the PGS was evaluated using 1000 Genomes European reference data rather than the study sample itself, as the modest sample size of the present cohort would have limited the precision and stability of LD estimates.

To reduce the risk of selection bias during polygenic score optimization, the false discovery rate (FDR) correction was applied to the *p*-values of each SNP using the Benjamini–Hochberg procedure. All four of the SNPs that were included in the final score (rs10772420, rs1527483, rs8065080, and rs1761667) remained statistically significant after correction (q < 0.05).

Given the modest sample size, we refrained from using internally estimated beta coefficients, as effect-size estimates derived from small samples are prone to sampling error and overfitting and may not generalize well to independent datasets. To avoid propagating potentially unstable weights, the PGS was constructed as an unweighted score, obtained by summing the number of risk alleles (0–2 per selected SNP), which is generally more robust in smaller cohorts.

For categorical analyses and trend testing, the total risk-allele count (PGS) was collapsed into five polygenic score groups: 1–3, 4, 5, 6, and 7–8 alleles.

### 2.9. Statistical Analysis and Covariate Selection

Statistical analyses were performed using the IBM Statistical Package for the Social Sciences (SPSS) version 26 (IBM Corp., Armonk, NY, USA) and Stata 17 (StataCorp LLC, College Station, TX, USA). Normality of continuous variables was assessed by the Kolmogorov–Smirnov test in SPSS; non-normal distributions were normalized by Templeton’s two-step transformation [45]. Categorical comparisons were conducted by χ^2^ tests, and continuous comparisons by Mann–Whitney U tests. Trends across polygenic score and folate categories were examined by the Jonckheere–Terpstra test [46]. The discriminative performance of the PGS for folate categories (<7, 7–13, and >13 µmol/L) was evaluated by receiver operating characteristic (ROC) curve analysis, with area under the curve reported. To account for multiple testing, false discovery rate (FDR) correction was applied to SNP-level *p*-values using the Benjamini–Hochberg procedure.

Preliminary analyses tested C-reactive protein (CRP) as a potential covariate to account for systemic inflammation and gastrointestinal pathology affecting folate absorption. However, CRP was not significantly associated with serum folate levels (*p* > 0.05), and its inclusion did not meaningfully improve model fit (ΔR^2^ < 0.01). Therefore, CRP was not included in the primary multivariable models. Alcohol consumption frequency was retained in all models based on known biological effects on folate metabolism. Commonly used medications were also included to control for potential medication effects.

Multivariable linear and logistic regression models were fitted in SPSS. Model I included age, sex, BMI, cardiometabolic parameters (systolic blood pressure, fasting glucose level, triglyceride level, and HbA1C), lifestyle (current smoking status and frequency of alcohol consumption), mental health (GHQ12 Likert) covariates, and genetic predictors (individual SNPs or polygenic score). Model II was Model I with an additional adjustment for the frequency of vegetable consumption. Robust (Huber–White) standard errors were obtained in Stata. Model fit was judged by adjusted R^2^ for linear models and Hosmer–Lemeshow tests for logistic models.

Interaction terms (PGS × vegetable intake and PGS × education) were introduced into fully adjusted models to test moderation effects. Mediation and moderated-mediation pathways were evaluated in Stata by generalized structural equation modeling (GSEM) with 1 000 bootstrap replications to derive 95% confidence intervals.

Type I error was controlled using the Benjamini–Hochberg false discovery rate (FDR) procedure applied to all SNP-level and SNP × taste preference interaction *p*-values (q < 0.05). Bonferroni correction was additionally performed as a sensitivity check but was not used to determine SNP inclusion in subsequent models. For the multivariable analyses, a stepwise linear regression was applied with SNPs entering the model at *p* < 0.05 and retained at *p* < 0.10. This procedure resulted in the selection of four SNPs for inclusion in the polygenic score.

Multicollinearity was assessed by variance inflation factors (<5). Missing data (<5% per variable) were handled via listwise deletion; sensitivity to missingness was verified by multiple imputation in SPSS.

## 3. Results

### 3.1. Basic Characteristics of Study Population

Participants were stratified into three groups based on their serum folate levels: <7 µmol/L, 7–13 µmol/L, and >13 µmol/L.

Individuals with higher serum folate levels tended to be older (mean age: 44.08 years, 95% CI: 42.82–45.34; *p* = 0.008) and to have higher BMI values (mean: 27.81 kg/m^2^, 95% CI: 27.21–28.40; *p* < 0.001). Fasting glucose levels also increased with folate concentration (mean: 5.21 mmol/L, 95% CI: 5.06–5.36; *p* = 0.002), while there was no significant trend in systolic blood pressure (*p* = 0.211). Psychological well-being, as assessed via the GHQ Likert scale, improved with increasing folate levels (mean score: 10.42, 95% CI: 9.96–10.89; *p* = 0.033). For more details, see Table 2.

Sociodemographic trends revealed a decreasing proportion of participants of Roma ethnicity (from 63.33% to 45.32%; *p* = 0.001) and of individuals with only primary education (from 63.33% to 47.85%; *p* < 0.001) as folate levels increased. Conversely, the proportion of participants with a college or university education rose significantly (from 3.33% to 12.15%). Smoking prevalence decreased with higher folate levels (from 60.20% to 42.03%; *p* < 0.001), while there was no significant trend in alcohol consumption frequency (*p* = 0.201). Use of antihypertensive medication increased significantly across folate categories (*p* = 0.021), whereas use of lipid-lowering medication did not differ significantly (*p* = 0.278). Use of antidiabetic medication showed a modest but significant increase (*p* = 0.045). For more details, see Table 2.

No significant trends were observed in the prevalence of indifference or dislike towards sweet, fatty, or salty foods; bitter chocolate; coffee without sugar; or various raw vegetables (e.g., kohlrabi, cabbage, cauliflower), with *p*-values ranging from 0.079 to 0.941. Preference for grapefruit also showed no significant association with folate levels (*p* = 0.261). In contrast, the frequency of vegetable consumption demonstrated a statistically significant trend (*p* = 0.035). Fruit consumption showed a borderline trend (*p* = 0.080). For more details, see Supplementary Table S1.

### 3.2. Associations Between Taste-Related SNPs and Serum Folate Levels

We examined the association between selected SNPs and serum folate concentrations. In Model I, two SNPs (rs10772420—A and rs1527483—A) showed statistically significant positive associations with serum folate levels (*p* < 0.05). The effect of rs10772420 remained significant in Model II (*p* = 0.003), while the association of rs1527483 was marginal (*p* = 0.092). SNP rs8065080 showed borderline significance in both models (Model I: *p* = 0.053; Model II: *p* = 0.066). No significant associations were observed for the remaining SNPs in either model. For more details, see Table 3.

### 3.3. Polygenic Score Association with Folate Levels

PGS was constructed using taste-related SNPs to evaluate their cumulative association with serum folate levels. Stepwise inclusion based on individual regression coefficients and model fit (Δ*p*-value and R^2^) identified four SNPs (rs10772420—A, rs1527483—A, rs8065080—C, and rs1761667—A) as contributors to the optimized score. Each of the included SNPs showed a statistically significant positive association with folate levels (e.g., rs10772420: β = 1.178, 95% CI: 0.401–1.955, *p* = 0.003), and incremental improvements in explained variance were observed (final model R^2^ = 0.196). SNPs with weaker or inconsistent effects (rs713598, rs307355, rs239345, and rs2274333) were excluded from the final score. All four of the SNPs included in the final score remained statistically significant after FDR correction (q < 0.05), confirming their robust association with serum folate levels. See Table 4 for more details.

LD analysis based on 1000 Genomes European reference data confirmed low correlation between rs10772420 and rs1761667 (R^2^ = 0.032) and negligible LD among all other SNP pairs.

The PGS constructed was a significant predictor of serum folate status in the logistic regression model. Individuals with higher PGS values had increased odds of having folate levels above the >13 µmol/L threshold, with an odds ratio of 1.208 (95% CI: 1.049–1.390, *p* = 0.009).

Participants were stratified into five groups based on their PGS, ranging from low (1–3) to high (7–8). A clear positive trend was observed between increasing PGS and serum folate concentration. Individuals in the lowest PGS group (1–3) had an average folate level of 14.17 nmol/L (95% CI: 12.83–15.52), whereas those in the highest group (7–8) exhibited significantly higher levels (19.47 nmol/L, 95% CI: 17.00–21.93). The trend across groups was statistically significant (*p* for trend < 0.001), indicating a dose–response relationship between genetic predisposition and folate status.

Similarly, the prevalence of optimal folate levels increased with higher PGS. In the lowest group, 54.32% (95% CI: 43.48–64.86) of individuals had optimal folate levels, compared to 71.43% (95% CI: 59.50–81.44) in the highest group. This association was also statistically significant (*p* for trend = 0.003), supporting the predictive utility of the polygenic score in identifying individuals with favorable folate profiles. For more details, see Supplementary Table S2.

To evaluate the discriminative ability of the PGS in identifying individuals with folate deficiency, ROC curve analysis was performed. Although statistically significant, the AUC of 0.569 (95% CI: 0.524–0.614) indicates limited discriminative ability of the polygenic score in identifying folate deficiency.

### 3.4. Genotype–Phenotype Associations and Behavioral Modulation: Interaction, Mediation, and Moderated Effects

**Figure 1:**
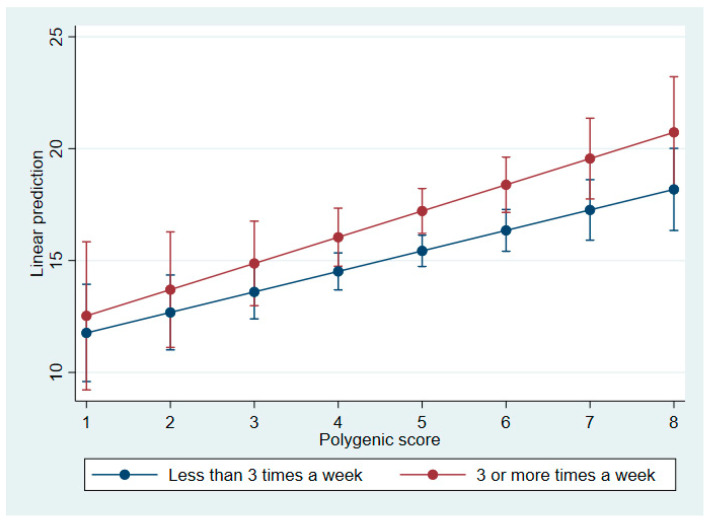
Predicted serum folate concentrations by polygenic score and vegetable intake frequency. This plot shows the adjusted regression lines for serum folate across PGS values, stratified by vegetable consumption: solid line: ≥3 servings/week; dashed line: <3 servings/week.

Regression models revealed a significant interaction between PGS and vegetable consumption (β = 0.36; *p* = 0.003). Marginal effects analysis indicated that individuals with frequent vegetable intake exhibited a steeper increase in serum folate levels across rising PGS values (see Figure 1). A similar interaction was observed between PGS and educational attainment, with stronger genetic effects among individuals with vocational or higher education (β range = 0.33–0.56; *p* < 0.05). No significant interaction was found between PGS and metabolic parameters (e.g., glucose, triglycerides, HbA1C; all *p* > 0.3).

**Figure 2:**
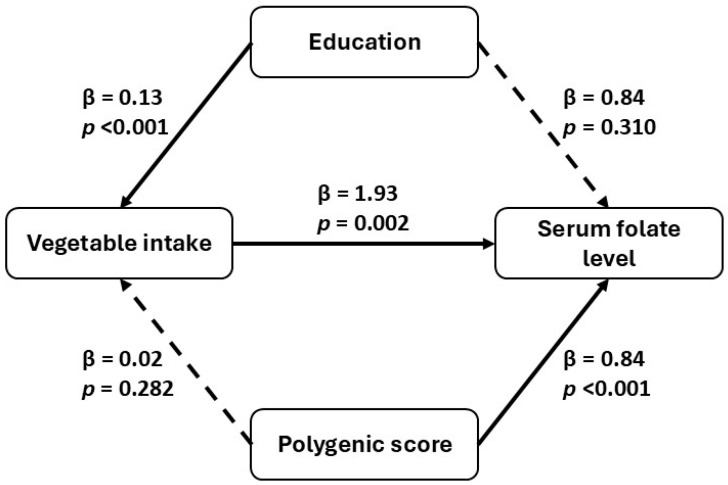
Path diagram of direct and indirect associations of polygenic score, education and frequency of vegetable intake on serum folate levels. Significant paths are indicated by solid arrows (standardized β and *p*-values), while non-significant paths are indicated by dashed arrows.

Structural equation modeling examined associations between education, PGS, and serum folate levels, revealing patterns consistent with potential mediation through vegetable consumption. Education exerted an association on folate (β = 0.84, *p* = 0.310) and a significant indirect association via vegetable consumption (β = 0.13, *p* < 0.001), which itself strongly predicted folate levels (β = 1.93, *p* = 0.002). This indirect pathway accounted for approximately 30.8% of education’s total effect. PGS showed a robust direct association with folate (β = 0.84, *p* < 0.001), while its indirect path through vegetable intake was weaker and not statistically significant (β = 0.02, *p* = 0.282). These findings highlight the behavioral mediation of educational attainment and the primarily direct influence of genetic predisposition on folate status. See Figure 2 for more details.

Moderated mediation analyses showed that the indirect association of PGS via vegetable intake did not significantly differ by sex or ethnicity. Specifically, the subgroup-specific indirect associations were negligible and statistically non-significant (e.g., females: β = −0.00052, *p* = 0.846; Roma ethnicity: β = 0.011, *p* = 0.856; Hungarian: β = −0.057, *p* = 0.578), indicating no meaningful moderation.

Ethnicity-stratified models revealed that vegetable intake significantly predicted serum folate levels among Hungarian general individuals (β = 2.32, *p* = 0.010), whereas the association was weaker and non-significant in the Roma ethnic subgroup (β = 1.17, *p* = 0.149), suggesting potential cultural or behavioral differences in dietary mediation.

## 4. Discussion

The aim of this study was to investigate how taste-related genetic variants, aggregated into a polygenic score, together with dietary behavior and sociodemographic factors, modulate serum folate levels and contribute to interindividual variability in Hungarian general and Roma ethnic adult populations. Our findings highlight a previously underexplored pathway linking bitter-taste sensitivity to folate status, which is independent of vegetable intake.

The *TAS2R19* rs10772420 variant was found to be the strongest and most consistent predictor of serum folate levels in our cohort. Notably, this association remained statistically significant even after adjusting for vegetable intake (β = 6.24 nmol/L, *p* = 0.016), indicating a robust association rather than behavioral mediation. Although TAS2R19 is a bitter taste receptor and allelic variation has been linked to avoidance of bitter-tasting foods in several populations, the persistence of the association after dietary adjustment suggests that the mechanism in our cohort is unlikely to be fully explained by food selection behavior. Nevertheless, heightened bitter sensitivity may influence long-term dietary patterns in subtle ways, such as reduced preference for bitter vegetables, coffee, or certain fruits, which could contribute to micronutrient status over time. The cross-sectional design, however, does not allow us to disentangle behavioral from physiological pathways.

Our results are consistent with those of previous studies by Lucock et al. [26] and Calancie et al. [47], who reported similar genotype–phenotype associations in Australian and US populations, respectively. Importantly, the present study builds upon earlier research conducted in the same Hungarian cohort (Diószegi et al. [38]), where *TAS2R19* genotypes were shown to shape vegetable consumption patterns in both Hungarian and Roma minority subgroups. By linking taste receptor variation not only to dietary behavior but also to biochemical folate status, our findings extend the behavioral–genetic paradigm and underscore the relevance of sensory genetics in micronutrient epidemiology.

The PGS, which was constructed using four taste-related SNPs (*TAS2R19* rs10772420, *OR10G4* rs1527483, *TRPV1* rs8065080, and *CD36* rs1761667), showed a robust and statistically significant association with serum folate levels. Each included variant contributed positively to folate status, and the cumulative score exhibited a clear dose–response trend, with individuals in the highest PGS group (7–8) showing markedly higher folate concentrations than those in the lowest group (1–3).

Importantly, the association between PGS and folate remained significant even after adjusting for vegetable intake, consistent with a genetic association not substantially mediated by self-reported vegetable consumption. This is consistent with emerging evidence on extra-oral expression of taste receptors, including *TAS2R19* and *TRPV1*, in gastrointestinal and hepatic tissues, which may influence nutrient absorption and metabolic regulation independently of dietary behavior [48,49].

Regression models revealed a significant interaction between PGS and vegetable intake (β = 0.36; *p* = 0.003). Individuals with frequent vegetable consumption exhibited a steeper increase in folate levels as PGS values rose, indicating a synergistic effect between genetic predisposition and dietary behavior. Although vegetable intake did not mediate the association between bitter taste-related genetic variants and folate levels, it significantly moderated the effect of the polygenic score. This interaction suggests that genetic predisposition may be amplified or attenuated depending on habitual vegetable consumption.

Stronger genetic effects were observed among individuals with vocational or higher education, consistent with a significant interaction between PGS and education. This suggests that educational attainment may enhance the phenotypic expression of genetic predisposition, potentially via improved dietary literacy or access.

Structural equation modeling further clarified these pathways: while education influenced folate status both directly and indirectly via vegetable intake, the PGS primarily exerted an association, with its indirect path through vegetable consumption remaining non-significant. These findings support a dual-pathway model in which behavioral and genetic factors independently contribute to micronutrient status, in line with the nutrigenomic framework described by Wagner-Reguero et al. [50].

Sociodemographic factors, particularly educational attainment, emerged as powerful modifiers. Higher educational attainment was associated with increased folate levels and amplified genetic effects, suggesting that health literacy or epigenetic mechanisms may enhance the expression of genetic predisposition. Vegetable intake mediated a portion of the education-folate relationship, reinforcing the role of dietary behavior as a bridge between social determinants and biological outcomes. The observed mediation underscores not only individual behavior but also the role of environmental context. Importantly, food environments are modifiable through policy or community-level interventions, such as introducing subsidized vegetable markets, establishing urban gardens, or expanding culturally adapted produce programs, which may reinforce positive dietary behaviors in socially disadvantaged groups. These findings are consistent with those of Krishnaswamy and Nair [51], who emphasized the role of socioeconomic status and education in shaping folate intake and status, particularly in non-fortified populations.

Ethnicity-stratified analyses confirmed the association between rs10772420 and folate in both the Hungarian general and Roma ethnic populations, but vegetable-mediated effects were significant only in the Hungarian general cohort. This divergence likely reflects cultural and environmental differences in food availability, taste preferences, and health behaviors [52,53]. Prior studies have shown that bitter-sensitive individuals in marginalized communities may lack access to palatable vegetable alternatives, reinforcing dietary avoidance. Addressing these barriers requires culturally sensitive interventions. Our previous complex health survey [27] revealed a high prevalence of metabolic syndrome and insulin resistance in both groups, with particularly unfavorable lipid profiles among Roma minority individuals. These metabolic disparities may interact with micronutrient status and warrant targeted nutritional interventions. These findings underscore the need for culturally tailored nutritional strategies that consider genetic sensitivity and environmental constraints in underserved populations.

This study has several limitations that should be acknowledged. Firstly, the cross-sectional design precludes causal inference. While we describe associations and patterns consistent with mediation pathways, these reflect correlational evidence rather than true causal mechanisms. Our results should be interpreted as describing statistical associations in cross-sectional data rather than as consistent with a potential pathway. Longitudinal or Mendelian randomization approaches would be necessary to establish causality and directionality. Secondly, the PGS was constructed solely from taste-related SNPs, excluding key metabolic variants such as *MTHFR* and *FOLH1*, which are known to influence folate absorption and utilization. This limited genomic scope may partly explain the modest predictive performance of the PGS. Additionally, the polygenic score was derived using a simple allele-count model without effect-size weighting, which may limit its comparability with polygenic scores constructed from genome-wide association studies (GWAS). Future research should consider standardization procedures to improve transferability and predictive calibration across populations. Thirdly, dietary behavior was assessed via self-reported questionnaires, which are subject to recall bias and social desirability effects. Fourthly, serum folate reflects short-term dietary intake (half-life: 2–3 weeks) and may not represent chronic folate status captured by red blood cell (RBC) folate, reflecting tissue stores accumulated over 3–4 months [54]. This single-time-point measurement may introduce misclassification bias, particularly in individuals with episodic dietary patterns, and may be influenced by acute factors such as inflammation or recent supplementation. Additionally, serum folate was measured using chemiluminescent microparticle immunoassay (CMIA), which may yield different values compared to microbiological assays, limiting comparability across studies [55]. Concurrent measurement of both serum and RBC folate using standardized assay methodologies would have improved assessment of long-term folate status and cross-study comparability [56]. Finally, while the sample was representative of the Northeast Hungarian population, generalizability to other regions or ethnic groups may be constrained.

Despite these limitations, the study has several notable strengths. Notably, it is one of the first studies to integrate taste receptor genetics and polygenic scoring in the context of micronutrient status. The use of structural equation modeling and moderated mediation analyses provides a nuanced understanding of behavioral and social pathways. The inclusion of both the Hungarian general and the Roma ethnic populations enhances the cultural relevance and equity focus of the findings. These methodological innovations meaningfully contribute to the emerging fields of nutrigenetics and precision public health.

From a public health perspective, these findings support the development of personalized dietary guidance for non-fortified populations. Education emerged as a key leverage point, suggesting that improving health literacy could amplify genetic benefits. Compared to fortified populations in North America or Asia, the Hungarian cohort represents a unique dietary and genetic landscape. The observed associations may differ in settings with mandatory folic acid fortification, underscoring the importance of tailoring nutrigenomic strategies to local nutritional environments. Genome-based personalized nutrition technologies offer promising avenues for precision health, especially in populations with high metabolic risk and limited access to fortified foods. Our results contribute to this emerging field by highlighting the sensory–genetic dimension of micronutrient status and its relevance for targeted prevention.

## 5. Conclusions

This study demonstrates that taste-related genetic variation, particularly bitter-taste sensitivity mediated by the *TAS2R19* gene, is significantly associated with serum folate concentrations in a non-fortified Hungarian adult population. The effect persists independently of vegetable intake and is further shaped by sociodemographic factors such as educational attainment. The polygenic score, constructed from four taste-related SNPs, exhibited a clear dose–response relationship with folate levels, although its standalone predictive capacity for deficiency remains modest.

By extending previous research conducted in the same population, our findings reinforce the role of sensory genetics in shaping dietary behavior and micronutrient status. Ethnicity-stratified analyses revealed cultural and environmental differences in gene–diet interactions, underscoring the need for tailored nutritional strategies, particularly for vulnerable groups such as the Roma ethnic population, who experience disproportionately high rates of metabolic syndrome and insulin resistance.

Although the cross-sectional design limits causal inference, the study provides a robust foundation for future longitudinal and Mendelian randomization research. Incorporating metabolic gene variants and leveraging machine learning approaches may enhance predictive accuracy and support the development of personalized nutrition models.

In the broader context of nutrigenetics, these findings highlight the importance of integrating sensory perception, genetic predisposition, and social determinants into dietary guidance. In non-fortified settings such as Hungary, genome-informed nutrition strategies, coupled with targeted education and culturally adapted interventions, may offer promising avenues for improving micronutrient intake and reducing health disparities.

## Figures and Tables

**Table 1:** Selected SNPs associated with taste perception and dietary preferences.

SNP ID	Gene	Chromosome Position	Literature-Based Relevance
rs10772420	*TAS2R19*	7q34	Bitter taste perception; associated with vegetable intake and folate-rich food preferences [34]
rs1527483	*OR10G4*	11q24.2	Smell sensitivity; may influence food choices and micronutrient intake [35]
rs1761667	*CD36*	7q21.11	Fat taste perception; linked to lipid metabolism and metabolic syndrome [36,37]
rs2274333	*TAS2R16*	7q31.1	Bitter taste receptor; associated with alcohol and vegetable consumption [38,39]
rs239345	*TAS2R9*	12p13.2	Bitter taste receptor; may affect dietary preferences [40]
rs307355	*OR2J3*	6q23.2	Odor sensitivity; potential role in food selection behavior [40]
rs713598	*TAS2R38*	7q34	Bitter taste perception; associated with vegetable intake and folate-rich food preferences [41]
rs8065080	*TRPV1*	17p13.2	Heat/pungency perception; may influence spicy food intake and dietary folate indirectly [42]

**Table 2:** Sociodemographic, clinical, and lifestyle characteristics across serum folate level categories. *: *p* < 0.05, statistically significant.

		Groups Based on Serum Folate Levels			*p* for Trend
		<7 µmol/L (Low; n = 30)	7–13 µmol/L (Normal; n = 201)	>13 µmol/L (Optimal; n = 395)	
		Average (95% CI)			
Age (years)		40.73 (36.50–44.97)	41.63 (40.03–43.24)	44.08 (42.82–45.34)	0.008 *
BMI (kg/m^2^)		25.36 (22.91–27.82)	26.32 (25.55–27.10)	27.81 (27.21–28.40)	<0.001 *
Systolic blood pressure (mmHg)		119.80 (114.26–125.34)	125.19 (123.01–127.37)	125.83 (124.21–127.45)	0.211
Fasting glucose level (mmol/L)		4.78 (4.30–5.26)	4.98 (4.75–5.21)	5.21 (5.06–5.36)	0.002 *
Triglyceride level (mmol/L)		1.44 (1.17–1.72)	1.63 (1.46–1.79)	1.50 (1.41–1.59)	0.686
HbA1C level (%)		5.45 (5.27–5.63)	5.48 (5.39–5.57)	5.53 (5.46–5.60)	0.805
GHQ Likert scale		11.50 (9.32–13.68)	11.23 (10.52–11.93)	10.42 (9.96–10.89)	0.033
		Average in % (95% CI)			*p* for trend
Women		70.00 (52.35–84.00)	68.16 (61.49–74.30)	64.30 (59.49–68.91)	0.289
Roma ethnicity		63.33 (45.51–78.72)	57.71 (50.81–64.39)	45.32 (40.46–50.24)	0.001 *
Education	Primary	63.33 (45.51–78.72)	62.69 (55.86–69.15)	47.85 (42.95–52.7)	<0.001 *
	Vocational/High School	33.33 (18.60–51.11)	32.34 (26.16–39.02)	40.00 (35.26–44.89)	
	College/University	3.33 (0.36–14.54)	4.98 (2.59–8.65)	12.15 (9.21–15.65)	
Current smoker		53.33 (35.87–70.19)	60.20 (53.33–66.78)	42.03 (37.23–46.94)	<0.001 *
Alcohol consumption	Less than 1 time a month	56.67 (39.01–73.11)	51.74 (44.85–58.58)	49.11 (44.21–54.04)	0.201
	1 time a month	36.67 (21.28–54.49)	33.83 (27.56–40.57)	32.66 (28.17–37.40)	
	More than 1 time a month	6.67 (1.41–19.71)	14.43 (10.09–19.78)	18.23 (14.66–22.26)	
Use of antihypertensive medication		20.00 (8.81–36.66)	25.37 (19.74–31.71)	33.16 (28.66–37.92)	0.021 *
Use of lipid-lowering medication		16.67 (6.66–32.74)	9.95 (6.38–14.66)	8.61 (6.14–11.68)	0.278
Use of antidiabetic medication		3.33 (0.36–14.54)	5.97 (3.30–9.89)	10.13 (7.44–13.39)	0.045 *

Values are presented as mean (95% CI) or n (%). *p* < 0.05 for trend across serum folate categories. BMI: body mass index; CI: confidence interval; GHQ: General Health Questionnaire; HbA1c: glycated hemoglobin.

**Table 3:** Association between individual SNPs and serum folate concentration (linear regression analysis).

		Model I.		Model II.	
SNP ID	Effect Allele	B (95% CI)	*p*-Value	B (95% CI)	*p*-Value
rs10772420	A	1.050 (0.280–1.820)	0.008 *	1.118 (0.403–1.958)	0.003 *
rs1527483	A	1.561 (0.079–3.044)	0.039 *	1.317 (−0.217–2.852)	0.092
rs8065080	C	0.793 (−0.011–1.597)	0.053	0.760 (−0.051–1.571)	0.066
rs1761667	A	0.431 (−0.348–1.210)	0.278	0.437 (−0.356–1.229)	0.280
rs713598	C	0.238 (−0.496–0.971)	0.525	0.212 (−0.532–0.956)	0.576
rs307355	C	0.296 (−0.996–1.589)	0.653	0.127 (−1.173–1.427)	0.848
rs239345	A	0.180 (−0.758–1.118)	0.706	0.033 (−0.919–0.986)	0.945
rs2274333	A	0.148 (−0.642–0.938)	0.714	0.108 (−0.693–0.909)	0.791

Model I: adjusted for age, sex, Roma ethnicity, education level, systolic blood pressure, fasting glucose, triglycerides, HbA1C, smoking status, alcohol consumption, GHQ12 score, and antihypertensive, antidiabetic, and lipid-lowering treatment. Model II: Model I was further adjusted for the frequency of vegetable consumption. *: *p* < 0.05, statistically significant.

**Table 4:** Stepwise inclusion of SNPs for polygenic score optimization based on association with serum folate levels. Ref.: first reference SNP included in the stepwise method.

SNP ID	Polygenic Score				Included/ Excluded
	B (95% CI)	*p*-Value	Change in *p*-Value	R^2^	
rs10772420	1.178 (0.401–1.955)	3.02 × 10^−3^	Ref.	0.187	Included
rs1527483	1.235 (0.552–1.919)	4.18 × 10^−4^	−2.60 × 10^−3^	0.192	Included
rs8065080	1.039 (0.519–1.559)	9.72 × 10^−5^	−3.21 × 10^−4^	0.196	Included
rs1761667	0.838 (0.421–1.255)	9.03 × 10^−5^	−6.94 × 10^−6^	0.196	Included
rs713598	0.684 (0.323–1.046)	2.23 × 10^−4^	1.32 × 10^−4^	0.194	Excluded
rs307355	0.764 (0.366–1.162)	1.80 × 10^−4^	9.02 × 10^−5^	0.194	Excluded
rs239345	0.705 (0.328–1.082)	2.65 × 10^−4^	1.74 × 10^−4^	0.193	Excluded
rs2274333	0.687 (0.310–1.064)	3.70 × 10^−4^	2.79 × 10^−4^	0.192	Excluded

## Data Availability

Due to data protection and ethical concerns, the dataset(s) supporting the conclusions of this article are available upon request from the study coordinators, Róza Ádány (adany.roza@med.unideb.hu) and Péter Pikó (piko.peter@med.unideb.hu).

## Supplementary Materials

The following supporting information can be downloaded at https://www.mdpi.com/article/10.3390/nu18040562/s1, Table S1: Preferences and consumption frequency by serum folate levels categories (n = 626); Table S2: Dose–response association between polygenic score (PGS) groups and serum folate concentration (µmol/L) and prevalence (%) of optimal folate status (>13 µmol/L).

## Abbreviations

The following abbreviations are used in this manuscript:

ALT	Alanine Aminotransferase
ALP	Alkaline Phosphatase
AUC	Area Under the Curve
BMI	Body Mass Index
CI	Confidence Interval
CRP	C-Reactive Protein
DNA	Deoxyribonucleic Acid
FDR	False Discovery Rate
GGT	Gamma-Glutamyl Transferase
GHQ	General Health Questionnaire
GWAS	Genome-Wide Association Study
HbA1C	Hemoglobin A1C
HDL-C	High-Density Lipoprotein Cholesterol
HWE	Hardy–Weinberg Equilibrium
LDL-C	Low-Density Lipoprotein Cholesterol
MTHFR	Methylenetetrahydrofolate Reductase
NTD	Neural Tube Defect
PGS	Polygenic Score
ROC	Receiver Operating Characteristic
SEM	Structural Equation Modeling
SNP	Single-Nucleotide Polymorphism
SPSS	Statistical Package for the Social Sciences
TG	Triglyceride
TRPV1	Transient Receptor Potential Vanilloid 1

## References

[1] Crider K.S., Yang T.P., Berry R.J., Bailey L.B. (2012). Folate and DNA methylation: A review of molecular mechanisms and the evidence for folate’s role. Adv. Nutr..

[2] Mc Auley M.T., Mooney K.M., Salcedo-Sora J.E. (2016). Computational modelling folate metabolism and DNA methylation: Implications for understanding health and ageing. Brief. Bioinform..

[3] Viswanathan M., Urrutia R.P., Hudson K.N., Middleton J.C., Kahwati L.C. (2023). Folic Acid Supplementation to Prevent Neural Tube Defects: Updated Evidence Report and Systematic Review for the US Preventive Services Task Force. JAMA.

[4] de la Fournière B., Dhombres F., Maurice P., de Foucaud S., Lallemant P., Zérah M., Guilbaud L., Jouannic J.-M. (2020). Prevention of Neural Tube Defects by Folic Acid Supplementation: A National Population-Based Study. Nutrients.

[5] Barua S., Kuizon S., Junaid M.A. (2014). Folic acid supplementation in pregnancy and implications in health and disease. J. Biomed. Sci..

[6] Mattson M.P., Haberman F. (2003). Folate and homocysteine metabolism: Therapeutic targets in cardiovascular and neurodegenerative disorders. Curr. Med. Chem..

[7] Bottiglieri T., Laundy M., Crellin R., Toone B.K., Carney M.W., Reynolds E.H. (2000). Homocysteine, folate, methylation, and monoamine metabolism in depression. J. Neurol. Neurosurg. Psychiatry.

[8] Bottiglieri T. (1996). Folate, Vitamin B12, and Neuropsychiatric Disorders. Nutr. Rev..

[9] Tong H., Walker N. (2021). Current levels of coverage of iron and folic acid fortification are insufficient to meet the recommended intake for women of reproductive age in low- and middle-income countries. J. Glob. Health.

[10] Aydin S., Jenkins A., Detchou D., Barrie U. (2024). Folate fortification for spina bifida: Preventing neural tube defects. Neurosurg. Rev..

[11] Williams J., Mai C.T., Mulinare J., Isenburg J., Flood T.J., Ethen M., Frohnert B., Kirby R.S., Centers for Disease Control and Prevention (2015). Updated estimates of neural tube defects prevented by mandatory folic Acid fortification—United States, 1995–2011. Morb. Mortal. Wkly. Rep..

[12] Ray J.G. (2008). Efficacy of Canadian folic acid food fortification. Food Nutr. Bull..

[13] European Food Safety Authority (2009). ESCO Report on Analysis of Risks and Benefits of Fortification of Food with Folic Acid.

[14] EUROCAT (2007). Special Report on Periconceptional Folic Acid Supplementation.

[15] Liu S., West R., Randell E., Longerich L., O’Connor K.S., Scott H., Crowley M., Lam A., Prabhakaran V., McCourt C. (2004). A comprehensive evaluation of food fortification with folic acid for the primary prevention of neural tube defects. BMC Pregnancy Childbirth.

[16] Murphy M.E., Westmark C.J. (2020). Folic Acid Fortification and Neural Tube Defect Risk: Analysis of the Food Fortification Initiative Dataset. Nutrients.

[17] European Food Safety Authority (2014). Dietary Reference Values for Folate.

[18] Thuesen B.H., Husemoen L.L.N., Ovesen L., Jørgensen T., Fenger M., Linneberg A. (2010). Lifestyle and genetic determinants of folate and vitamin B12 levels in a general adult population. Br. J. Nutr..

[19] World Health Organization (2012). Serum and Red Blood Cell Folate Concentrations for Assessing Folate Status in Populations.

[20] Araszkiewicz A.F., Jańczak K., Wójcik P., Białecki B., Kubiak S., Szczechowski M., Januszkiewicz-Lewandowska D. (2025). MTHFR Gene Polymorphisms: A Single Gene with Wide-Ranging Clinical Implications—A Review. Genes.

[21] Cabo R., Hernes S., Slettan A., Haugen M., Ye S., Blomhoff R., Mansoor M.A. (2015). Effect of genetic polymorphisms involved in folate metabolism on the concentration of serum folate and plasma total homocysteine (p-tHcy) in healthy subjects after short-term folic acid supplementation: A randomized, double blind, crossover study. Genes Nutr..

[22] Cummings D., Dowling K.F., Silverstein N.J., Tanner A.S., Eryilmaz H., Smoller J.W., Roffman J.L. (2017). A Cross-Sectional Study of Dietary and Genetic Predictors of Blood Folate Levels in Healthy Young Adults. Nutrients.

[23] Diószegi J., Mohammad Kurshed A.A., Pikó P., Kósa Z., Sándor J., Ádány R. (2021). Association of single nucleotide polymorphisms with taste and food preferences of the Hungarian general and Roma populations. Appetite.

[24] Guo J., Xie H., Wang J., Zhao H., Wang F., Liu C., Wang L., Lu X., Bao Y., Zou J. (2013). The maternal folate hydrolase gene polymorphism is associated with neural tube defects in a high-risk Chinese population. Genes Nutr..

[25] Dioszegi J., Llanaj E., Adany R. (2019). Genetic Background of Taste Perception, Taste Preferences, and Its Nutritional Implications: A Systematic Review. Front. Genet..

[26] Lucock M., Ng X., Boyd L., Skinner V., Wai R., Tang S., Naylor C., Yates Z., Choi J.H., Roach P. (2011). TAS2R38 bitter taste genetics, dietary vitamin C, and both natural and synthetic dietary folic acid predict folate status, a key micronutrient in the pathoaetiology of adenomatous polyps. Food Funct..

[27] Ádány R., Pikó P., Fiatal S., Kósa Z., Sándor J., Bíró É., Kósa K., Paragh G., Bácsné Bába É., Veres-Balajti I. (2020). Prevalence of Insulin Resistance in the Hungarian General and Roma Populations as Defined by Using Data Generated in a Complex Health (Interview and Examination) Survey. Int. J. Environ. Res. Public Health.

[28] Széles G., Vokó Z., Jenei T., Kardos L., Pocsai Z., Bajtay A., Papp E., Pásti G., Kósa Z., Molnár I. (2005). A preliminary evaluation of a health monitoring programme in Hungary. Eur. J. Public Health.

[29] Kósa Z., Moravcsik-Kornyicki Á., Diószegi J., Roberts B., Szabó Z., Sándor J., Ádány R. (2015). Prevalence of metabolic syndrome among Roma: A comparative health examination survey in Hungary. Eur. J. Public Health.

[30] Piko P., Dioszegi J., Kosa Z., Sandor J., Moizs M., Adany R. (2021). Changes in the Prevalence of Metabolic Syndrome, Its Components, and Relevant Preventive Medication between 2011 and 2018 in the Northeast Hungarian Roma Population. J. Pers. Med..

[31] Piko P., Dioszegi J., Sandor J., Adany R. (2021). Changes in the Prevalence of Metabolic Syndrome and Its Components as Well as in Relevant Preventive Medication between 2006 and 2018 in the Northeast Hungarian Population. J. Pers. Med..

[32] O’Leary F., Samman S. (2010). Vitamin B12 in health and disease. Nutrients.

[33] Catanzaro D., Chesbro E.C., Velkey A.J. (2013). Relationship between food preferences and PROP taster status of college students. Appetite.

[34] Mikolajczyk-Stecyna J., Malinowska A.M., Chmurzynska A. (2020). Polymorphism of TAS2R3, TAS2R5, TAS2R19, and TAS2R50 genes and bitter food intake frequency inelderly woman. Acta Sci. Pol. Technol. Aliment..

[35] Jaime-Lara R.B., Brooks B.E., Vizioli C., Chiles M., Nawal N., Ortiz-Figueroa R.S.E., Livinski A.A., Agarwal K., Colina-Prisco C., Iannarino N. (2023). A Systematic Review of the Biological Mediators of Fat Taste and Smell. Physiol. Rev..

[36] Melis M., Carta G., Pintus S., Pintus P., Piras C.A., Murru E., Manca C., Di Marzo V., Banni S., Barbarossa I.T. (2017). Polymorphism rs1761667 in the CD36 Gene Is Associated to Changes in Fatty Acid Metabolism and Circulating Endocannabinoid Levels Distinctively in Normal Weight and Obese Subjects. Front. Physiol..

[37] Pepino M.Y., Kuda O., Samovski D., Abumrad N.A. (2014). Structure-function of CD36 and importance of fatty acid signal transduction in fat metabolism. Annu. Rev. Nutr..

[38] Kurshed A.A.M., Vincze F., Pikó P., Kósa Z., Sándor J., Adány R., Diószegi J. (2023). Taste Preference-Related Genetic Polymorphisms Modify Alcohol Consumption Behavior of the Hungarian General and Roma Populations. Genes.

[39] Subramanian G., Ponnusamy V., Vasanthakumar K., Panneerselvan P., Krishnan V., Subramaniam S. (2024). The gustin gene variation at rs2274333 and PROP taster status affect dietary fat perception: A stepwise multiple regression model study. J. Nutr. Biochem..

[40] Hejazi J., Amiri R., Nozarian S., Tavasolian R., Rahimlou M. (2024). Genetic determinants of food preferences: A systematic review of observational studies. BMC Nutr..

[41] Perna S., Riva A., Nicosanti G., Carrai M., Barale R., Vigo B., Allegrini P., Rondanelli M. (2018). Association of the bitter taste receptor gene TAS2R38 (polymorphism RS713598) with sensory responsiveness, food preferences, biochemical parameters and body-composition markers. A cross-sectional study in Italy. Int. J. Food Sci. Nutr..

[42] Forstenpointner J., Förster M., May D., Hofschulte F., Cascorbi I., Wasner G., Gierthmühlen J., Baron R. (2017). Short Report: TRPV1-polymorphism 1911 A>G alters capsaicin-induced sensory changes in healthy subjects. PLoS ONE.

[43] Choi S.W., O’Reilly P.F. (2019). PRSice-2: Polygenic Risk Score software for biobank-scale data. GigaScience.

[44] Dudbridge F. (2013). Power and Predictive Accuracy of Polygenic Risk Scores. PLoS Genet..

[45] Templeton G.F., Brian Pope M., Burney L.L. (2021). The Usefulness of the Two-Step Normality Transformation in Retesting Existing Theories: Evidence on the Productivity Paradox. Data Base Adv. Inf. Syst..

[46] Manning S.E., Ku H.C., Dluzen D.F., Xing C., Zhou Z. (2023). A nonparametric alternative to the Cochran-Armitage trend test in genetic case-control association studies: The Jonckheere-Terpstra trend test. PLoS ONE.

[47] Calancie L., Keyserling T.C., Taillie L.S., Robasky K., Patterson C., Ammerman A.S., Schisler J.C. (2018). Predisposition to Bitter Taste Associated with Differential Changes in Vegetable Intake in Response to a Community-Based Dietary Intervention. G3 Genes Genomes Genet..

[48] Chen K.J., Liang X.Y., Yi H.Y., Yu G.X., Wu Q. (2025). Ectopic taste receptors in animal physiology: Evolutionary conservation and functional diversification. Front. Cell Dev. Biol..

[49] Tuzim K., Korolczuk A. (2021). An update on extra-oral bitter taste receptors. J. Transl. Med..

[50] Wagner-Reguero S., Fernández L.P., Colmenarejo G., Cruz-Gil S., Espinosa I., Molina S., Crespo M.C., Aguilar-Aguilar E., Marcos-Pasero H., de la Iglesia R. (2025). Sweet Taste Receptors’ Genetic Variability in Advanced Potential Targets of Obesity. Nutrients.

[51] Krishnaswamy K., Madhavan Nair K. (2001). Importance of folate in human nutrition. Br. J. Nutr..

[52] Fernqvist F., Spendrup S., Tellström R. (2024). Understanding food choice: A systematic review of reviews. Heliyon.

[53] Jayasinghe S., Byrne N.M., Hills A.P. (2025). Cultural influences on dietary choices. Prog. Cardiovasc. Dis..

[54] Pfeiffer C.M., Caudill S.P., Gunter E.W., Osterloh J., Sampson E.J. (2005). Biochemical indicators of B vitamin status in the US population after folic acid fortification: Results from the National Health and Nutrition Examination Survey 1999–2000. Am. J. Clin. Nutr..

[55] Bailey R.L., McDowell M.A., Dodd K.W., Gahche J.J., Dwyer J.T., Picciano M.F. (2010). Total folate and folic acid intakes from foods and dietary supplements of US children aged 1–13 y. Am. J. Clin. Nutr..

[56] Pfeiffer C.M., Zhang M., Lacher D.A., Molloy A.M., Tamura T., Yetley E.A., Picciano M.F., Johnson C.L. (2011). Comparison of serum and red blood cell folate microbiologic assays for national population surveys. J. Nutr..

