# Supplementary material for: Folate Status Shaped by Taste Receptor Genetics and Sociobehavioral Modulation: Evidence from a Hungarian Cohort
Source: Nutrients. 2026 Feb 8;18(4):562. doi: 10.3390/nu18040562 (PMC12943046; doi:10.3390/nu18040562)
Supplement: Supplementary file 1 [file nutrients-18-00562-s001.zip › Sup. Table 1.pdf]

	Groups based on serum folate levels			
	<7 µmol/L (low; n = 30)	7–13 µmol/L (normal; n = 201)	>13 µmol/L (optimal; n = 395)	p-for trend
Food preference	Prevalence of indifferent or dislike in % (95%CI)			
Sweet foods	23.33 (11.09 – 40.44)	26.87 (21.10 – 33.29)	23.04 (19.09 – 27.38)	0.383
Fatty foods	50.00 (32.80 – 67.20)	40.80 (34.17 – 47.68)	39.49 (34.76 – 44.38)	0.451
Salty foods	40.00 (24.05 – 57.78)	43.78 (37.05 – 50.69)	41.01 (36.24 – 45.91)	0.624
Bitter chocolate	60.00 (42.22 – 75.95)	66.67 (59.95 – 72.91)	66.58 (61.82 – 71.10)	0.758
Coffee without sugar	96.67 (85.46 – 99.64)	88.06 (83.04 – 91.99)	87.34 (83.79 – 90.35)	0.377
Raw kohlrabi	46.67 (29.81 – 64.13)	62.69 (55.86 – 69.15)	59.24 (54.34 – 64.00)	0.941
Raw cabbage	66.67 (48.89 – 81.40)	74.13 (67.76 – 79.81)	65.82 (61.04 – 70.37)	0.079
Raw cauliflower	66.67 (48.89 – 81.40)	80.60 (74.72 – 85.60)	80.51 (76.39 – 84.18)	0.433
Grapefruit	73.33 (55.90 – 86.51)	67.66 (60.98 – 73.84)	64.30 (59.49 – 68.91)	0.261
Consumption	Prevalence of less than 3 times/week in % (95%CI)			p-for trend
Fruit	57.69 (38.73 – 75.04)	64.65 (57.81 – 71.05)	56.10 (51.12 – 61.00)	0.080
Vegetable	69.23 (50.20 – 84.25)	73.58 (67.04 – 79.41)	64.30 (59.40 – 68.99)	0.035*

Supplementary Table S1. Preferences and consumption frequency by serum folate levels categories (n = 626). Prevalence of indifference/dislike and low intake (<3 times/week) across folate strata; p-for trend values from Jonckheere–Terpstra test. *: $p < 0.05$, statistically significant
