# Supplementary material for: Folate Status Shaped by Taste Receptor Genetics and Sociobehavioral Modulation: Evidence from a Hungarian Cohort
Source: Nutrients. 2026 Feb 8;18(4):562. doi: 10.3390/nu18040562 (PMC12943046; doi:10.3390/nu18040562)
Supplement: Supplementary file 1 [file nutrients-18-00562-s001.zip › Sup. Table 2.pdf]

| PGS | Average folate levels in μmol/L (95%CI) | p for trend | Proportion of individuals (in%) with optimal folate status (95%CI) | p for trend |
|---|---|---|---|---|
| 1 – 3 | 14.17 (12.83 – 15.52) | <0.001* | 54.32 (43.48 – 64.86) | 0.003* |
| 4 | 14.80 (13.75 – 15.84) | | 58.33 (50.18 – 66.15) | |
| 5 | 15.77 (14.70 – 16.84) | | 62.63 (55.74 – 69.14) | |
| 6 | 16.32 (15.17 – 17.46) | | 70.00 (62.06 – 77.12) | |
| 7 – 8 | 19.47 (17.00 – 21.93) | | 71.43 (59.50 – 81.44) | |

**Supplementary Table S2.** Dose–response association between polygenic score (PGS) groups and serum folate concentration (μmol/L) and prevalence (%) of optimal folate status (>13 μmol/L). *: $p < 0.05$, statistically significant
